# Outcomes of Secondary Intracapsular Intraocular Lens Implantation in Patients following Rhegmatogenous Retinal Detachment †

**DOI:** 10.3390/jcm12247749

**Published:** 2023-12-18

**Authors:** Kaicheng Wu, Jiemei Shi, Yuan Zong, Gezhi Xu, Haohao Zhu, Chunhui Jiang

**Affiliations:** 1 Department of Ophthalmology and Vision Science, Eye and ENT Hospital, Fudan University, Shanghai 200031, China; 13301050254@fudan.edu.cn (K.W.); 15301050261@fudan.edu.cn (J.S.); zongyuan326@163.com (Y.Z.); xugezhi@sohu.com (G.X.); 2 Key Laboratory of Myopia of the State Health Ministry, Shanghai 200031, China; 3 Key Laboratory of Visual Impairment and Restoration of Shanghai, Shanghai 200031, China; 4 Department of Ophthalmology, Shanghai Fifth People’s Hospital, Fudan University, Shanghai 200240, China

**Keywords:** intracapsular IOL implantation, IOL position, refractive outcomes, secondary IOL implantation, vitrectomized eyes

## Abstract

This study reports the outcomes of a secondary IOL implantation technique in patients that suffered from rhegmatogenous retinal detachment combined with a cataract, which included reopening the capsular bag, enabling secondary intracapsular intraocular lens (IOL) implantation. We included consecutive cases with rhegmatogenous retinal detachment (RRD) treated with vitrectomy and silicone oil tamponade, and subsequent secondary IOL implantation during silicone oil removal between September 2019 and June 2022. Demographics, pre- and postoperative clinical data, and complications were collected. Visual and refractive outcomes and IOL position were evaluated. Thirty eyes were included and followed up for a mean of 24.2 ± 5.06 months. Compared with the preoperative values, no significant changes were observed in the intraocular pressure (*p* = 0.170) and endothelial cell density (*p* = 0.336); however, the best-corrected visual acuity (Snellen: 20/83 vs. 20/38; logMAR: 0.66 ± 0.23 vs. 0.37 ± 0.32; *p* < 0.001) and spherical equivalent *(p* < 0.001) improved significantly. The mean prediction error (ME) was −0.45 ± 0.68 D (−1.9–0.54 D), and the mean absolute prediction error (MAE) was 0.62 ± 0.52 D (0.01–1.9 D). The macula-on subgroup demonstrated significantly better refractive outcomes than the macula-off subgroup (ME, *p* = 0.046; MAE, *p* = 0.008). The IOL was well positioned, with a mean horizontal and vertical tilt and decentration of 0.53 ± 0.49° and 0.21 ± 0.16 mm, and 0.54 ± 0.45° and 0.22 ± 0.16 mm, respectively. Secondary intracapsular IOL implantation provided a good and stable IOL position and satisfactory refractive outcomes, and is a feasible treatment option for patients with RRD.

## 1. Introduction

Pars plana vitrectomy (PPV) combined with phacoemulsification are increasingly used in managing vitreoretinal diseases. Particularly, they are routinely performed in patients with rhegmatogenous retinal detachment (RRD) combined with a cataract [1,2,3]. Even if the cataract is not so heavy that it interferes with PPV, the combined phacoemulsification facilitates better removal of the vitreous base so as to achieve a high reattachment rate [4]. However, concomitant implantation of an intraocular lens (IOL) remains controversial. The first concern is that the IOL power cannot be determined precisely in patients with RRD, particularly the macula-off cases [5]. In a previous study, we found that the axial length (AL) might change after PPV with silicone oil tamponade, particularly in patients with hypotony and high myopia [6]. In addition, the intravitreal tamponade affects the IOL position. The intraocular tamponade reportedly pushes the IOL toward PPV, combined with phacoemulsification and IOL implantation, causing a myopic shift [7,8]. Moreover, the implanted IOL may interfere with the postoperative observation of the peripheral retina during the follow-up visit.

To maintain the advantages of PPV combined with phacoemulsification while minimizing the complications of simultaneous IOL implantation, we introduced a new technique of a secondary IOL implantation in the capsular bag. With this approach, the IOL implantation in the capsular bag is performed in a second act during the removal of silicone oil. The key to this technique is the reopening of the capsular bag, which was challenging [9]. Herein, we report the outcomes of the 2-year follow-up of this technique, focusing on the IOL position, the refractive outcomes, and the association between the postoperative refractive error and various clinical factors.

## 2. Materials and Methods

### 2.1. Patients and Study Design

This case series study included consecutive patients who suffered from RRD combined with a cataract and had already undergone phacoemulsification without IOL implantation, PPV, and silicone oil (Oxane 5700, Bausch + Lomb Inc., Bridgewater, NJ, USA)/air tamponade. The secondary intracapsular IOL implantation was performed during silicone oil removal between September 2019 and June 2022 at our hospital and followed up for a mean of 24.2 ± 5.06 months. The following were the exclusion criteria: (1) intraocular diseases other than cataract and RRD, (2) retinal re-detachment during follow-up, (3) history of previous vitreous or retinal procedure, (4) unsuccessful secondary intracapsular IOL implantation, and (5) follow-up duration of less than 6 months.

This study complied with the tenets of the Declaration of Helsinki and was approved by the Ethics Committee of the Eye and ENT Hospital. All patients provided informed consent.

### 2.2. Surgical Technique

The surgical technique used has been previously described in detail [10]. Briefly, during primary vitrectomy, continuous curvilinear capsulorhexis and phacoemulsification were performed, and the capsular bag was preserved to be intact. At the secondary surgery, after the silicone oil was removed, the anterior chamber (AC) was filled with a viscoelastic agent (DisCoVisc; Alcon Laboratories Inc., Fort Worth, TX, USA, or Medical Sodium Hyaluronate Gel; QISHENG, Shanghai, China), and capsular forceps were used to grasp the outer edge of the proliferation and pull centripetally, separating the proliferation from the anterior and posterior capsules. Next, a viscoelastic agent was injected into the capsular bag, into which an IOL (Rayner 920H, Rayner Intraocular Lenses Ltd., Hove, East Sussex, UK, or SA60AT, Alcon Laboratories Inc., Fort Worth, TX, USA) was subsequently implanted (see Video S1 in the electronic Supplementary Materials).

### 2.3. Peri- and Postoperative Data

The following data were collected: age, sex, relevant ocular and systemic history, and best-corrected visual acuity (BCVA), spherical equivalent (SE), intraocular pressure (IOP), corneal endothelial cell density, and slit-lamp microscopy and dilated fundus examination findings.

Preoperatively, patients underwent a detailed examination, including fundus check using a noncontact lens (Maxfield 84 Diopter; Ocular Instruments, Bellevue, WA, USA), best-corrected visual acuity (BCVA) assessment, intraocular pressure measurement (NT400, Nidek Corp., Ltd., Aichi, Japan), and corneal endothelial cell count (Topcon America Corp., Paramus, NJ, USA). Keratometric and axial length (AL) measurements were performed using IOLMaster 700 (version 3.01; Carl Zeiss AG, Jena, Germany). Based on the IOLMaster measurements, K values were calculated as 1/2 (K1 + K2).

Similarly, at 1, 6, and 24 months postoperatively, BCVA, SE, IOP, corneal endothelial cell density, and slit-lamp microscopy and dilated fundus examination findings were recorded. Furthermore, at each postoperative visit, the IOL position was measured on images at 90° and 180° obtained using Pentacam HR (Oculus Optikgeräte, Wetzlar, Germany) [11]. In addition, complications were documented.

### 2.4. IOL Power Calculation and Refractive Measurements

We calculated the IOL power and the predicted spherical equivalent (SE) preoperatively using biometric measurements (IOLMaster 700, version 3.01) and the SRK-T formula (http://www.eyecalcs.com/WEBCALCS/IOLcalc2/IOL2.html; accessed on the day of operation, and check again on 20 August 2023). To compare the refractive outcomes obtained using different formulas, the predicted SE was back-calculated using Emmetropia Verifying Optical (EVO, version 2.0, https://www.evoiolcalculator.com/calculator.aspx; accessed on 20 August 2023) and Barrett Universal II (BUII, version 1.05, http://calc.apacrs.org/barrett_universal2105/; accessed on 20 August 2023). Optimized A constants were downloaded from the User Group for Laser Interference Biometry website (www.ocusoft.de/ulib/c1.html; accessed on 20 August 2023).

The refractive prediction error (PE) was calculated as the postoperative SE (spherical power + 1/2 cylinder power) minus the preoperative predicted refraction. We calculated the mean error (ME), mean absolute error (MAE), and median absolute error (MedAE), which were the mean of all PEs and the mean and median of the absolute PEs, respectively.

To analyze whether the postoperative myopic shift in our cohort was associated with IOL movement, we collected data of another 30 age-, sex-, and AL-matched patients who underwent simple cataract removal and toric IOL implantation (Acrysof IQ SN6AT3-6, Alcon Laboratories Inc., Fort Worth, TX, USA) at our institution, and a comparison of the anterior chamber depth (ACD) at 2 months postoperatively between the two cohorts was performed.

### 2.5. Statistical Analysis

Data are presented as mean ± standard deviation for continuous variables and frequency (percentage) for categorical variables. The Kolmogorov–Smirnov test was adopted to identify the distribution of data. Student’s *t*-test (continuous variables) or the nonparametric chi-squared test (categorical variables) was used for comparison of the two cohorts. We compared the pre- and postoperative data using a paired *t*-test (normally distributed data) or Wilcoxon’s test (non-normally distributed data). Univariate linear regression was employed to identify risk factors for higher PEs. Friedman’s nonparametric test was used to compare the performance of the three formulas. Using the Cochran Q test, we compared the proportion of eyes that achieved PEs within ±0.25, ±0.50, and ±1.0 D. IBM SPSS Statistics (version 20.0, IBM Corp., Armonk, NY, USA) was used for all statistical analyses, and statistical significance was set at *p* < 0.05.

## 3. Results

In total, 30 eyes of 30 patients (18 males and 12 females) were included. The mean age was 55.77 ± 10.32 (range, 25–74) years. The demographic data of the included patients are presented in Table S1 in the electronic Supplementary Materials. The mean AL was 24.88 ± 2.02 mm (range, 22.24–29.01 mm). The mean K value was 43.41 ± 1.77 D (range, 38.29–46.35 D). Rayner 920H and Alcon SA60AT IOLs were implanted in 17 eyes (56.67%) and 13 eyes (43.33%), respectively.

After a mean follow-up of 24.2 ± 5.06 months (range, 13–36 months), compared with the preoperative values, the BCVA significantly increased (Snellen, 20/83 vs. 20/38; LogMAR, 0.66 ± 0.23 vs. 0.37 ± 0.32; *p* < 0.001) and the SE significantly improved (9.07 ± 3.42 vs. (−)2.32 ± 2.09; *p* < 0.001); however, no significant changes were observed in the intraocular pressure and endothelial cell count (Table 1). During the follow-up, posterior capsular opacification occurred in five patients (16.67%). All of them received Nd:YAG laser treatment.

At the final visit, after a mean follow-up duration of 24.2 ± 5.06 months, the mean horizontal and vertical IOL tilt and decentration were 0.53 ± 0.49° and 0.21 ± 0.16 mm, and 0.54 ± 0.45° and 0.22 ± 0.16 mm, respectively. For the 22 patients who completed the 2 years follow-up visit, there was no significant change in the IOL position during the follow-up period at 6 months and 2 years (Table 2).

**Figure 1:**
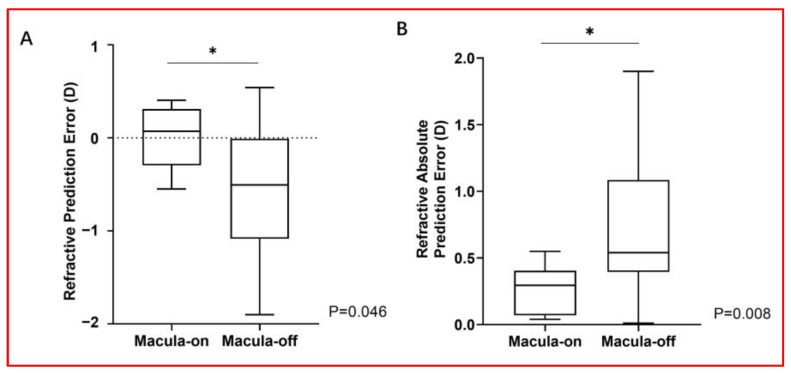
Refractive prediction error (**A**) and the refractive absolute prediction error (**B**) in the macula-on and macula-off group. * *p* < 0.05, statistically significant.

**Figure 2:**
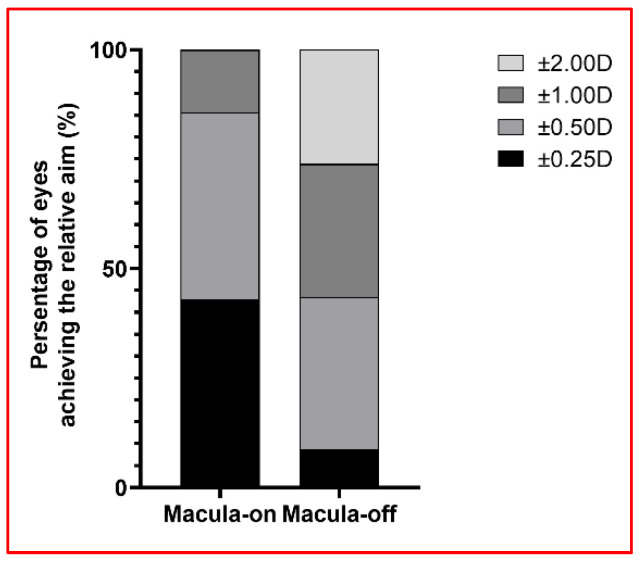
Proportion of eyes achieving the refractive aim.

Regarding refractive outcomes, an overall mild myopic shift was observed. The ME was −0.45 ± 0.68 D (range, (−)1.9–0.54 D), the MAE was 0.62 ± 0.52 D (range, 0.01–1.9 D), and the MedAE was 0.465 D. Interestingly, the refractive outcomes significantly differed according to the macular status before PPV, with patients in the macula-on subgroup demonstrating significantly better outcomes than those in the macula-off subgroup (ME, *p* = 0.046; MAE, *p* = 0.008) (Figure 1). ME and MAE were 0.00 ± 0.33 D and 0.25 ± 0.19 D in seven patients in the macula-on subgroup, respectively, and (−)0.58 ± 0.70 D and 0.73 ± 0.53 D in those in the macula-off subgroup, respectively. Patients in the macula-on subgroup had a higher percentage of PE within ±0.25 D (42.9% vs. 8.7%, *p* = 0.068), ±0. 50 D (85.7% vs. 43.5%, *p* = 0.125), and ±1.00D (100% vs. 73.9%, *p* = 0.170) than those in the macula-off subgroup (Figure 2). No significant differences were found in the refractive outcomes according to the IOL type (ME, *p* = 0.205; MAE, *p* = 0.509; see Table S2 in the electronic Supplementary Materials for details). The refractive PE was within ±1.00 D in 24 (80%) eyes. All six eyes with a PE greater than ±1.00 D had macula-off RRD (Figure 2). The univariate analysis revealed that the refractive PE was significantly associated with the macular status before PPV (on or off; *p* = 0.046), but not with other clinical factors, including ACD before PPV, retinal detachment duration, silicone oil tamponade duration, AL before silicone oil removal, IOL type (Rayner 920H or SA60AT), or the IOP change before and after silicone oil removal (Table 3). Regarding the refractive outcomes, three IOL calculation formulas were compared. The MAE, MedAE, and the proportion of cases with a PE ≤ 1.00 D were similar regardless of the calculation formula used (SRK/T, Barret Universal II, or EVO; Table 4). Although SRK/T showed the lowest ME (−0.45 ± 0.68 D) in comparison to the other two (BUII: −0.46 ± 0.70 D; EVO: −0.52 ± 0.64 D), the BUII formula displayed the highest percentage of the postoperative refractive error within ±0.25 D (26.7%) than the other two (SRK/T: 16.7%; EVO: 23.3%). The MedAE, in order of lowest to highest, was BUII (0.423 D), EVO (0.45 D), and SRK/T (0.465 D).

## 4. Discussion

We previously developed a technique for separating the adherent capsular bag in vitrectomized aphakic eyes and enabling secondary intracapsular IOL implantation [10]. In this study, we found that this technique provided a good and stable IOL position and satisfactory refractive outcomes.

RRD is the most common form of retinal detachment, and its incidence varies with age, peaking at sixty years old [12]. Therefore, most patients with RRD have age-related cataracts. Moreover, cataracts may develop within 1–3 years in patients after vitrectomy [13]. Even in those without heavy cataracts, performing phacoemulsification simultaneously ensures better visualization during retinal surgery and improves the cleaning of the vitreous base, providing more thorough tamponade and a more complete intraoperative laser treatment [4]. The PPV performed on the phakic eye will induce progressive cataract development and a rapid need for cataract surgery. For the silicone oil tamponade eyes, cataracts were formed in almost all eyes where oil was left in place for over 3–4 months. Although the lens was clear during silicone oil removal, 60% developed visually significant cataracts within 2 years [14,15,16,17]. For the gas tamponade eyes, a recent study reported that the median time from PPV to the indication of cataract extraction surgery was 19.0 months [6,18]. Moreover, performing phacoemulsification in vitrectomized eyes is more challenging owing to the lack of vitreous support [4,19]. Thus, lens removal is usually performed, and the capsular bag is preferably left intact to preserve the lens/iris septum and reduce the risk of silicone oil or gas entering the AC [20]. However, IOL implantation performed concurrently with PPV has been reported to induce a postoperative myopic shift [21], along with an increased IOL tilt and decentration [7]. Alternatively, the IOL can be implanted in the ciliary sulcus during a second operation; however, this is also associated with a refractive shift [22,23], a relatively high IOL tilt and decentration [24], and pupillary capture of the IOL optics [25]. Reopening the capsular bag for secondary intracapsular IOL implantation is a third option, which, as shown in this study, results in a good and stable IOL position and satisfactory refractive outcomes.

The in-the-bag IOL implantation was recommended for providing the greatest IOL stability and for placing it closer to the physiological, anatomical position of the original crystalline lens than the AC (angle supported) implantation, retropupillary iris/claw fixation, ciliary sulcus implantation, or trans-scleral fixation [22]. However, in PPV combined with phacoemulsification for RRD cases with intraocular tamponade, gas, or silicone oil had increased the IOL tilt and decentration [7]. Moreover, the duration of the silicone oil tamponade was positively associated with the extent of the IOL tilt and decentration [26]. The mean IOL tilt and decentration in this study of approximately 0.5° and 0.2 mm, respectively, are comparable to those after an uneventful phacoemulsification [27] and better than those in eyes with an IOL fixation in the ciliary sulcus [24]. The IOL tilt and decentration can cause astigmatism, coma, and higher-order aberrations that lower visual performance, particularly for aspheric IOLs, such as the Rayner 920H used in our study [28,29]. Aspheric IOLs have been shown to lose the advantage in visual quality with a decentration of >0.4 mm and a tilt of >7° [30]. Our outcomes were better, with an intraocular astigmatism [31] of only −0.12 ± 0.65 D. In addition, the results at 6 months and 2 years postoperatively indicated a stable IOL position.

We identified an overall mild myopic shift in the refractive outcomes: ME of −0.45 ± 0.68 D and MAE of 0.62 ± 0.52 D. The MAE was comparable to that reported in cases treated with sequential phacoemulsification and IOL implantation surgery [32,33], and better than that reported in cases of IOL implantation during PPV [5,32,33,34]. We further analyzed the factors that might be associated with the myopic shift, and found a significant association with the macular status before PPV; that is, the macula-off RRD cases tended to achieve a greater postoperative refractive error, similar to the findings of Moussa et al. [32]. Notably, there was a high percentage of macula-off cases (23/20, 76.67%) in this study, which may interfere with the refractive outcomes. Considering only the macula-on subgroup, the ME and MAE were 0.00 ± 0.33 D and 0.25 ± 0.19 D, respectively, comparable to those after an uneventful cataract surgery [35]. Although the mechanism of the macular status affecting the refractive error is unclear, the reduction in the retinal thickness [36] and decreased macular function [37] in macula-off RRD cases have been proposed to interfere with AL measurement [38]. The mild myopic shift observed in this and previous studies [33,39] also suggested that in previously macula-off RRD cases, a refractive target of +0.45 D should be adopted when performing a secondary IOL implantation.

Many new formulas were recently created. The BUII formula was regarded as the most accurate and predictable [40,41], while the EVO formula, developed by Tun Kuan Yeo, could achieve better results without ACD [42]. Similarly, previous findings indicated that the limitation of the SRK/T formula may account for the relatively anterior position of the effective lens position [21]. Thus, in this study, we included the outcomes of the BUII and EVO with no ACD in the analysis. Then, we compared them with the traditional formula. i.e., SRK/T. Previously, Zhang et al. suggested that newer formulas might be more accurate than traditional formulas, such as SRK/T [21]. However, in our study, ME and MAE values obtained using BUII and EVO were similar to those obtained using SRK/T. However, this result may be affected by the limited cases in this study because validating the formula might require more cohort data.

Previous studies have indicated that in the cases of PPV combined with phacoemulsification, IOL implantation, and the intravitreal tamponade, the capsular bag containing the IOLs would be pushed forward during the tamponade stage. Moreover, since the capsular bag is in the fibrosis process, even after the removal or absorption of the intravitreal tamponade, the capsular bag containing the IOL would not return to the original position, and the forward movement would be permanently preserved [43,44]. To test whether silicone oil affected the position of the capsular bag in this study, we compared the postoperative ACD in our cohort with that in characteristic-matched patients who underwent simple cataract surgery. Unexpectedly, we found that the ACD after a secondary intracapsular IOL implantation was similar to that after simple cataract surgery at 2 postoperative months (4.81 ± 0.63 mm vs. 4.93 ± 0.40 mm, *p* = 0.478; see Table S3 in the electronic Supplementary Materials for details). The reason is unclear. A self-control study could be conducted, where the position of the posterior capsular bag should be continuously monitored after the use of the silicone oil tamponade to clarify whether the silicone oil would push a capsule bag not containing an IOL forward. Similarly, the role of anterior IOL movement should be explored.

In this study, posterior capsular opacification occurred in five eyes during the follow-up. After laser treatment, the BCVA improved in all eyes. In another three eyes, a capsular tear occurred during the second procedure, and IOL fixation in the ciliary sulcus was performed. These three cases were not included in this study. However, the small number of cases with capsular tears suggest a high success rate of the surgical technique. Moreover, IOLs could be successfully implanted, even in eyes with complications, such as capsular ruptures.

More recently, we expanded the application of the novel technique to five patients with RRD who were treated with air tamponade. They underwent a similar secondary IOL implantation. Table S4 shows the results. After an average follow-up period of 5.20 ± 0.84 months, the BCVA (LogMAR, pre 0.54 ± 0.25, post 0.40 ± 0.34, *p* = 0.280), IOP (pre 14.84 ± 2.08 mmHg, post 17.02 ± 3.39 mmHg, *p* = 0.387), and ECD (pre 2576 ± 252.86 cells/mm^2^, post 2354 ± 347.74 cells/mm^2^, *p* = 0.177) remained unchanged. The SE improved from (+)9.00 ± 2.93 D to −2.38 ± 2.05 D (*p* = 0.000). At the last follow-up visit, the mean horizontal and vertical IOL tilt and decentration were 1.01 ± 0.80° and 0.50 ± 0.36 mm, and 0.72 ± 0.57° and 0.49 ± 0.39 mm, respectively. The ME was −0.51 ± 0.36 D (range from (−)1.04 to (−)0.045 D). These initial results reveal that the technique of secondary IOL implantation may be applied in more surgeries, given its safety and effectiveness. However, among the five air tamponade cases, only one achieved a PE within ±0.25 D. Therefore, whether gas and silicone oil tamponades affect the postoperative myopic shift differently needs further large-scale studies with longer follow-up periods.

There were some limitations to this study. First, this study did not set a real control group, that is, a simultaneous IOL implantation during retinal detachment surgery. In addition, the cases were limited to 30 eyes, and 22 participants completed the 2-year follow-up. Moreover, some challenges need further investigation, such as the mechanism by which the macular status affects the refractive error and the differences in the effect of silicone oil or air tamponade on the capsular bag position. Therefore, further randomized controlled studies should be designed, with an enlarged cohort and a prolonged follow-up time, to further confirm the efficacy and safety of the secondary intracapsular IOL implantation technique.

## 5. Conclusions

The secondary intracapsular IOL implantation technique provided an alternative time option for IOL implantation in RRD cases with silicone oil or air tamponade. The 2-year follow-up results showed a stable IOL position and an overall mild myopic shift, which was associated with the macular status. Therefore, the clinical application of this technique could be further explored.

## Figures and Tables

**Table 1:** Comparison of clinical parameters before and after surgery.

Parameters	Preoperative	Postoperative	*p* Value
BCVA (Snellen)	20/83	20/38	
BCVA (LogMAR)	0.66 ± 0.23	0.37 ± 0.32	<0.001 *
SE	(+)9.07 ± 3.42	(−)2.32 ± 2.09	<0.001 *
IOP (mmHg)	16.25 ± 3.29	17.05 ± 3.68	0.170
ECD (cells/mm^2^)	2477.83 ± 321.87	2405.50 ± 295.53	0.336

IOP, intraocular pressure; ECD, endothelial cell density; BCVA, best-corrected visual acuity; LogMAR, logarithm of the minimum angle of resolution; SE, spherical equivalent. * The paired *t*-test (normally distributed data) or Wilcoxon’s test (non-normally distributed data) was used to compare the pre- and postoperative clinical data. *p* < 0.05 indicated statistical significance.

**Table 2:** IOL position at the 6-month and the 2-year follow-up (N = 22).

IOL Position	Tilt (°)			Decentration (mm)		
	At 6 Months	At 2 Years	*p* Value	At 6 Months	At 2 Years	*p* Value
Horizontal	0.51 ± 0.30	0.47 ± 0.37	0.544	0.22 ± 0.24	0.19 ± 0.16	0.661
Vertical	0.46 ± 0.45	0.50 ± 0.40	0.372	0.23 ± 0.22	0.23 ± 0.15	0.758

IOL, intraocular lens. The paired *t*-test (normally distributed data) or Wilcoxon’s test (non-normally distributed data) was used to compare the data between the two time points.

**Table 3:** Association between the postoperative refractive error and clinical factors.

Variable	Univariate Linear Regression		
	Regression Coefficient	R^2^	*p* Value
Pre-PPV macular status (on, 0; off, 1)	−0.578	0.134	0.046 *
Pre-PPV ACD (mm)	−0.009	0.000	0.976
Retinal detachment duration (days)	−0.019	0.087	0.114
Silicone oil tamponade duration (months)	0.007	0.001	0.885
Pre-silicone oil removal AL (mm)	0.025	0.006	0.690
IOL type (Rayner 920H, 0; SA60AT, 1)	0.321	0.057	0.205
Pre- vs. post-silicone oil removal IOP change	0.012	0.003	0.771

Pre-PPV, before pars plana vitrectomy for rhegmatogenous retinal detachment; ACD, anterior chamber depth; AL, axial length; IOL, intraocular lens; IOP, intraocular pressure. * Univariate linear regression analysis was used. *p* < 0.05 indicated statistical significance.

**Table 4:** Refractive outcomes with the three different formulas.

	SRK/T	Barrett Universal II	EVO	*p* Value
MedAE	0.465	0.423	0.45	
MAE	0.62 ± 0.52	0.62 ± 0.56	0.63 ± 0.53	0.811 *
ME	−0.45 ± 0.68	−0.46 ± 0.70	−0.52 ± 0.64	0.188 *
±0.25 D	5 (16.7%)	8 (26.7%)	7 (23.3%)	0.417 †
±0.50 D	16 (53.3%)	16 (53.3%)	17 (56.7%)	0.846 †
±1.00 D	24 (80%)	24 (80%)	25 (83.3%)	0.368 †
±2.00 D	30 (100%)	29 (96.7%)	29 (96.7%)	0.368 †

MedAE, median absolute prediction error; MAE, mean absolute prediction error; ME, mean prediction error. * The Friedman nonparametric test was used to compare the prediction error and absolute prediction error among the three formulas. † The Cochran Q test was performed to compare the percentages of eyes with IOL prediction error within ±0.25, ±0.50, and ±1.0 D of the target refraction.

## Data Availability

All study-relevant data are included in this article or are uploaded as additional files.

## Supplementary Materials

The following supporting information can be downloaded at: https://www.mdpi.com/article/10.3390/jcm12247749/s1. Table S1. Demographic data of the included patients (Table, DOCX). Table S2. Refractive outcomes according to the macular status and IOL type (Table, DOCX). Table S3. Comparison of the baseline characteristics and 2-month postoperative ACD between patients who underwent secondary IOL implantation and those who underwent simple cataract surgery with primary IOL implantation (Table, DOCX). Table S4. Outcome of 5 cases underwent air tamponade for RRD and secondary IOL implantation (Table, DOCX). Video S1. Video demonstrating the surgical procedure. MP4.

## List of Abbreviations

ACD	Anterior chamber depth
AL	Axial length
BCVA	Best-corrected visual acuity
IOL	Intraocular lens
PPV	Pars plana vitrectomy (PPV)
MAE	Mean absolute error
ME	Mean error
MedAE	Median absolute error
PE	Prediction error
RRD	Rhegmatogenous retinal detachment
SE	Spherical equivalent
