# Supplementary material for: Outcomes of Secondary Intracapsular Intraocular Lens Implantation in Patients following Rhegmatogenous Retinal Detachment
Source: J Clin Med. 2023 Dec 18;12(24):7749. doi: 10.3390/jcm12247749 (PMC10743941; doi:10.3390/jcm12247749)
Supplement: Supplementary file 1 [file jcm-12-07749-s001.zip › Electronic supplementary material- Tables.pdf]

Table S1. Demographics of patients who underwent secondary intracapsular IOL implantation (N=30)

Parameters	Value
Sex	
Male	18
Female	12
Eye	
Right	18
Left	12
Mean age (years)	55.77±10.32
Mean AL (mm)	24.88±2.02
Mean K value (D)	43.41±1.77
IOL	
Rayner 920H	17
SA60AT	13
Mean IOL power (D)	19.47±3.67
Follow-up duration (months)	24.2±5.06

Data are presented as numbers or means ± standard deviations. AL, axial length; IOL, intraocular lens; K: keratometry.

Table S2. Refractive outcomes by macular status and IOL type

Refractive outcome (SRK/T)	ME (D)	MAE (D)
All eyes	-0.45±0.68	0.62±0.52
Macular status		
Macula-on (n=7)	-0.00±0.33	0.25±0.19
Macula-off (n=23)	-0.58±0.70	0.73±0.53
P value	0.046*	0.008*
IOL type		
Rayner 920H	-0.58±0.64	0.68±0.53
SA60AT	-0.26±0.71	0.54±0.51
P value	0.205	0.509

Data are presented as numbers or means ± standard deviations. IOL, intraocular lens; ME, mean prediction error; MAE, mean absolute prediction error.

*Student's t-test (normally distributed data) or nonparametric Mann-Whitney's test (non-normally distributed data) was used for subgroup comparisons. $P<0.05$ indicated statistical significance.

Table S3. Comparison of baseline characteristics and 2-month postoperative ACD in secondary versus primary IOL implantation

Parameters	Secondary intracapsular IOL implantation (N=30)	Control (phaco and primary IOL implantation) (N=30)	*P* value *
**Sex**			
**Female**	12	15	
**Male**	18	15	0.604
**Eye**			
**Right**	18	20	
**Left**	12	10	0.789
**Mean age (years)**	55.77±10.32	58.43±16.10	0.449
**Mean AL (mm)**	24.88±2.02	25.56±1.80	0.175
**Postoperative ACD (mm)**	4.81±0.63	4.93±0.40	0.478

Data are presented as numbers or mean ± standard deviation. IOL, intraocular lens; AL, axial length; ACD, anterior chamber depth.

*Student's t-test (for continuous variables) or chi-squared test (for categorical variables) was used for comparison between the two cohorts. $P<0.05$ indicated statistical significance

Table S4. Outcome of 5 cases underwent air tamponade for RRD and secondary IOL implantation

**Case**	**Gender/ Eye/ Age/**	**BCVA (LogMAR)**		**SE (D)**		**IOP (mmHg)**		**ECD (cells/mm2)**		**SRK/TME (D)**	**H-Decentration (mm)**	**H-Tilt (degree)**	**V-Decentration (mm)**	**V-Tilt (degree)**	**Follow-up (months)**
		Pre	Post	Pre	Post	Pre	Post	Pre	Post						
		P=0.280		*P*=0.000		*P*=0.387		*P*=0.177							
**1**	**M/L/53**	0.52	0.15	5.125	-4.375	14.4	15	2730	2283	-0.045	0.61	0.81	0.12	0.51	6
**2**	**M/L/58**	0.15	0.05	9.25	-4.75	18.5	13	2804	2630	-1.04	0.22	0.78	0.81	0.41	5
**3**	**F/L/70**	0.70	0.30	12.625	-0.625	13.6	19.9	2469	1859	-0.425	0.24	1.02	0.17	0.07	6
**4**	**F/L/65**	0.52	0.70	10.75	-0.5	13.5	16.1	2183	2262	-0.46	0.36	0.16	0.35	1.19	4
**5**	**M/L/55**	0.82	0.82	7.25	-1.625	14.2	21.1	2695	2740	-0.585	1.09	2.33	0.99	1.44	5
**Mean ± SD**		0.54 ± 0.25	0.40 ±0.34	9.00 ±2.93	-2.38 ±2.05	14.84 ±2.08	17.02 ±3.39	2576 ±252.86	2354 ±347.74	-0.51 ±0.36	0.50 ±0.36	1.01 ±0.80	0.49 ±0.39	0.72 ±0.57	5.20 ±0.84
